# Dietary n-3 polyunsaturated fatty acids, fish consumption, and endometrial cancer risk: a meta-analysis of epidemiological studies

**DOI:** 10.18632/oncotarget.18295

**Published:** 2017-05-30

**Authors:** Rui Hou, Shen-Shen Yao, Jia Liu, Lian-Lian Wang, Lang Wu, Luo Jiang

**Affiliations:** ^1^ Department of Obstetrics and Gynecology, Shengjing Hospital Affiliated to China Medical University, Shenyang, P.R. China; ^2^ Division of Epidemiology, Department of Medicine, Vanderbilt Epidemiology Center, Vanderbilt-Ingram Cancer Center, Vanderbilt University Medical Center, Nashville, Tennessee, USA; ^3^ Department of Ultrasound, Shengjing Hospital Affiliated to China Medical University, Shenyang, P.R. China

**Keywords:** n-3 fatty acids, fish, epidemiology, endometrial cancer

## Abstract

The relationship between intake of fish and n-3 fatty acids and endometrial cancer risk has not been consistent across epidemiological studies. We quantitatively assessed the aforementioned association through a systematic review and meta-analysis. PubMed and Embase were searched through March 2017 for eligible epidemiological studies. Fixed or random-effects models were used to pool relative risks (RRs) and 95% confidence intervals (CIs). The dose-response relationship was also evaluated. Based on the literature search, five prospective studies and 11 case-control studies were identified. All 16 studies were categorized as high-quality studies. After pooling available risk estimates, no significant association was detected between overall fish intake and endometrial cancer risk. In subgroup analyses, every one additional serving/week of fish intake was significantly associated with inversed endometrial cancer risk in studies adjusted for smoking (RR (95% CI): 0.95 (0.91–1.00)), or studies performed in Europe (RR (95% CI): 0.90 (0.84–0.97)), but not in other tested subgroups. In studies conducted in Asia, there was significant positive association (RR (95% CI): 1.15 (1.10–1.21)). Regarding n-3 PUFA intake, marginally inverse associations of high EPA or DHA intake were detected (EPA: RR (95% CI) = 0.79 (0.61–1.04); DHA: RR (95% CI) = 0.85 (0.64–1.11)). Dose-response analyses suggested a significant nonlinear relationship between DHA intake and endometrial cancer risk (p: 0.04). Overall, this meta-analysis suggests that intake of n-3 PUFA may be inversely associated with endometrial cancer risk at some level of evidence, although the exact relationship, especially for fish intake, needs further characterization. Further well-designed studies are warranted.

## INTRODUCTION

As one of the most common female pelvic malignancies, endometrial cancer represents the sixth leading cause of cancer incidence in women worldwide in 2012 [1]. In US alone, it is expected that there will be approximately 61,380 new endometrial cancer cases and 10,920 estimated deaths in 2017 [2]. It is critical to better understand the etiology of this cancer, and identify appropriate interventions to decrease its public health burden. Fish, an important part of diets worldwide, has been shown to be relevant to multiple human diseases, including several types of cancer [3–12]. Research suggests that specific types of fish contain high levels of n-3 polyunsaturated fatty acids (PUFAs) (e.g., eicosapentaenoic acid (EPA) and docosahexanoic acid (DHA)), which can potentially exert anti-infiammatory and anti-carcinogenic effects [13]. Despite this knowledge, however, the association between intake of fish and n-3 PUFA with endometrial cancer risk has not been consistently reported across epidemiological studies. For example, in several studies, the highest category of fish or n-3 PUFA intake was significantly associated with a decreased endometrial cancer risk [14–17]. Fernandez et al. reported a dose-response relationship between fish consumption and decreased endometrial cancer risk [13]. However, such a significant association was not detected in other studies [16, 18–23]. In two other studies conducted in Asia, high fish consumption was detected to confer an increased risk of developing endometrial cancer [24, 25]. Different studies may vary regarding study designs, subject eligibilities, statistical analyses and sample sizes. It is thus critical to synthesize available evidence to better understand whether fish/n-3 PUFA may represent one possible strategy for endometrial cancer prevention.

Bandera et al. conducted a meta-analysis summarizing studies up to 2006 and identified no association between overall fish intake and endometrial cancer risk [26]. In this study, however, authors did not synthesize evidence for the associations of n-3 PUFA including EPA and DHA. Besides, since the conduction of this meta-analysis, multiple additional studies evaluating association of fish consumption have been published [14, 16, 18–20, 24, 27, 28]. To better characterize the relationship between intakes of fish/n-3 PUFA and endometrial cancer risk, we thus conducted a systematic review and meta-analysis of all available epidemiological studies up to March 2017. We also performed dose-response analyses to carefully assess potential dose-response relationship of the research question of interest.

## RESULTS

### Literature search and study characteristics

**Figure 1:**
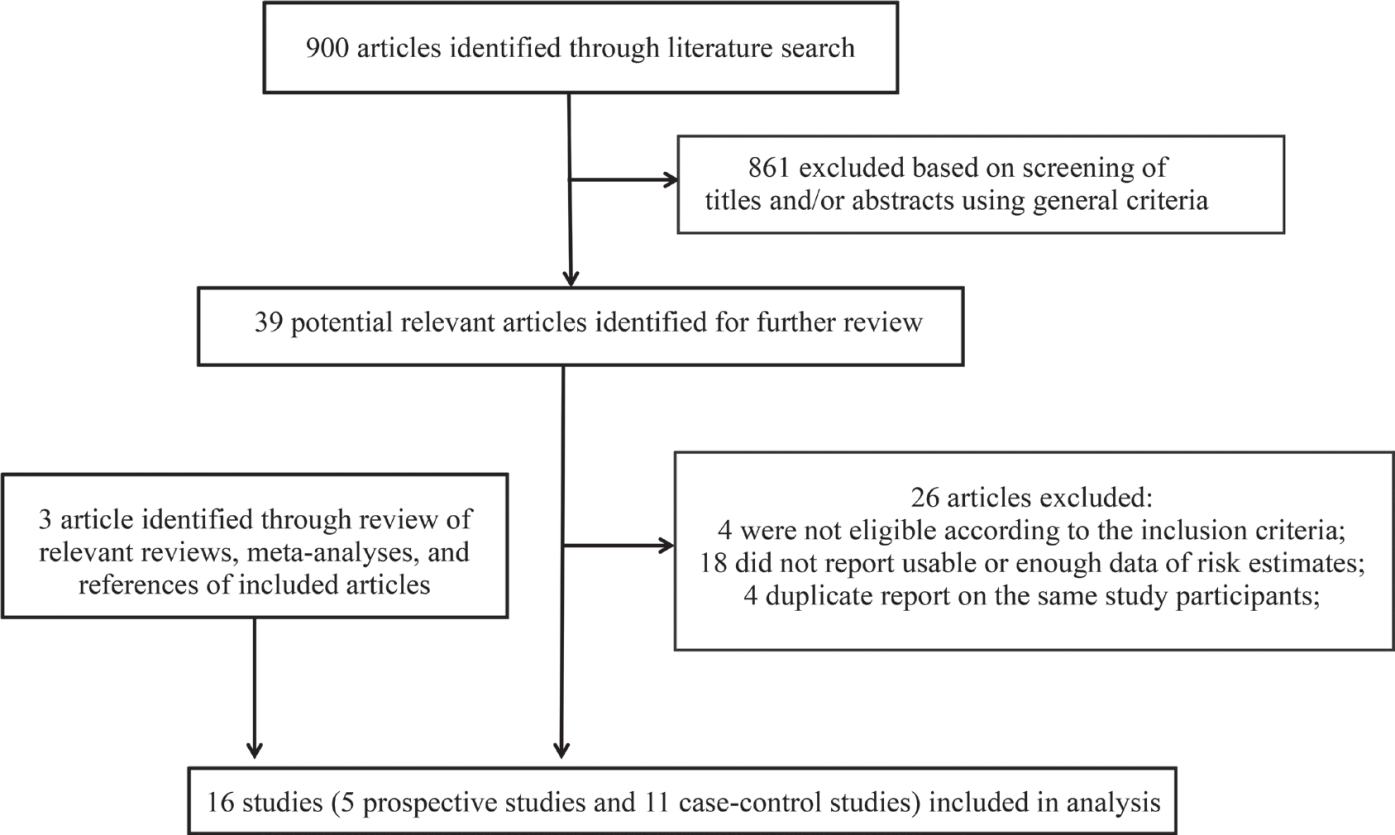
Flow chart for selection of eligible studies

The detailed literature search and article screening processes are shown in Figure 1. In brief, 900 articles were identified through the literature search. After screening the titles and abstracts using the predefined criteria, 861 articles were excluded, leaving 39 articles to be fully assessed. Among these articles, 26 were further excluded because they did not meet the eligibility criteria (*n* = 4), did not report usable data of risk estimates (*n* = 18), or contained duplicate subjects with other larger studies (*n* = 4). We further identified 3 additional eligible studies by screening the reference lists of the included studies and relevant review and meta-analysis publications. Overall, a total of 16 studies met our inclusion criteria and were included in the meta-analysis [13–22, 24, 25, 28–31]. The detailed characteristics of the included studies are shown in Supplementary Table 1. Briefly, four prospective cohort studies, one case-cohort study, and 11 case-control studies (including eight population-based case-control studies and three hospital-based case-control studies) were included. The prospective studies have relatively long follow-up periods (median 9–18 years; mean 6.5–9.1 years). The highest category of fish, DHA and EPA consumption ranged from > 1–2 servings/week to > 15.4 servings/week, 143 to 227 mg/d, and 74.7 to 127 mg/d, respectively. Nine were conducted in North America, four were conducted in Asia, and three were conducted in Europe. The quality ratings for these studies are shown in Tables 1 and 2. Overall, all 16 studies were classified as high-quality studies.

**Table 1:** Quality assessment of included prospective studies using the Newcastle-Ottawa Quality assessment scale*

Study	Exposed cohort represents average in community	Selection of the non-exposed cohort from same community	Ascertain exposure through records or structured interviews	Demonstrate that outcome not present at study start	Exposed and non-exposed matched and/or adjusted by factors	Ascertain outcome via independent blind assessment or record linkage	Follow-up long enough for outcome to occur	Loss to follow-up < 20%	Overall Score
Brasky, 2014	1	1	0	1	2	1	1	1	8
Brasky, 2016	1	1	0	1	2	1	1	1	8
Daniel, 2011	1	1	0	1	2	1	1	1	8
van Lonkhuijzen, 2011	1	1	0	1	2	1	1	1	8
Brasky, 2015	1	1	0	1	2	1	1	1	8

*A study could be awarded a maximum of one star for each item except for the item Control for important factor or additional factor. The definition/explanation of each column of the Newcastle-Ottawa Scale is available from (http://www.ohri.ca/programs/clinical_epidemiology/oxford.asp.). 1 means study adequately fulfilled a quality criterion (2 for exposed and non-exposed fully matched or adjusted by factors), 0 means it did not. Quality scale does not imply that items are of equal relevant importance

**Table 2:** Quality assessment of included case-control studies using the Newcastle-Ottawa Quality assessment scale*

Study	Case defined with independent validation	Representativeness of the cases	Selection of controls from community	Statement that controls have no history of outcome	Cases and controls matched and/or adjusted by factors	Ascertain exposure by blinded structured interview	Same method of ascertainment for cases and controls	Same response rate for both groups	Overall Score
Arem, 2013	0	1	1	1	2	0	1	1	7
Filomeno, 2015	1	1	0	1	2	1	1	1	8
Hirose, 1996	1	1	0	1	2	0	1	1	7
Takayama, 2013	1	1	1	1	2	0	1	1	8
Terry, 2002	1	1	1	0	2	0	1	1	7
Xu, 2006	1	1	1	0	2	1	1	1	8
McCann, 2000	1	1	1	0	2	1	1	1	8
Jain, 2000	1	1	1	0	2	1	1	0	7
Shu, 1993	1	1	1	0	1	1	1	1	7
Goodman, 1997	1	1	1	0	2	1	1	1	8
Fernandez, 1999	1	1	0	1	2	1	1	1	8

*A study could be awarded a maximum of one star for each item except for the item Control for important factor or additional factor. The definition/explanation of each column of the Newcastle-Ottawa Scale is available from (http://www.ohri.ca/programs/clinical_epidemiology/oxford.asp.). 1 means study adequately fulfilled a quality criterion (2 for case-control fully matched and adjusted), 0 means it did not. Quality scale does not imply that items are of equal relevant importance

### Fish consumption and endometrial cancer risk

**Figure 2:**
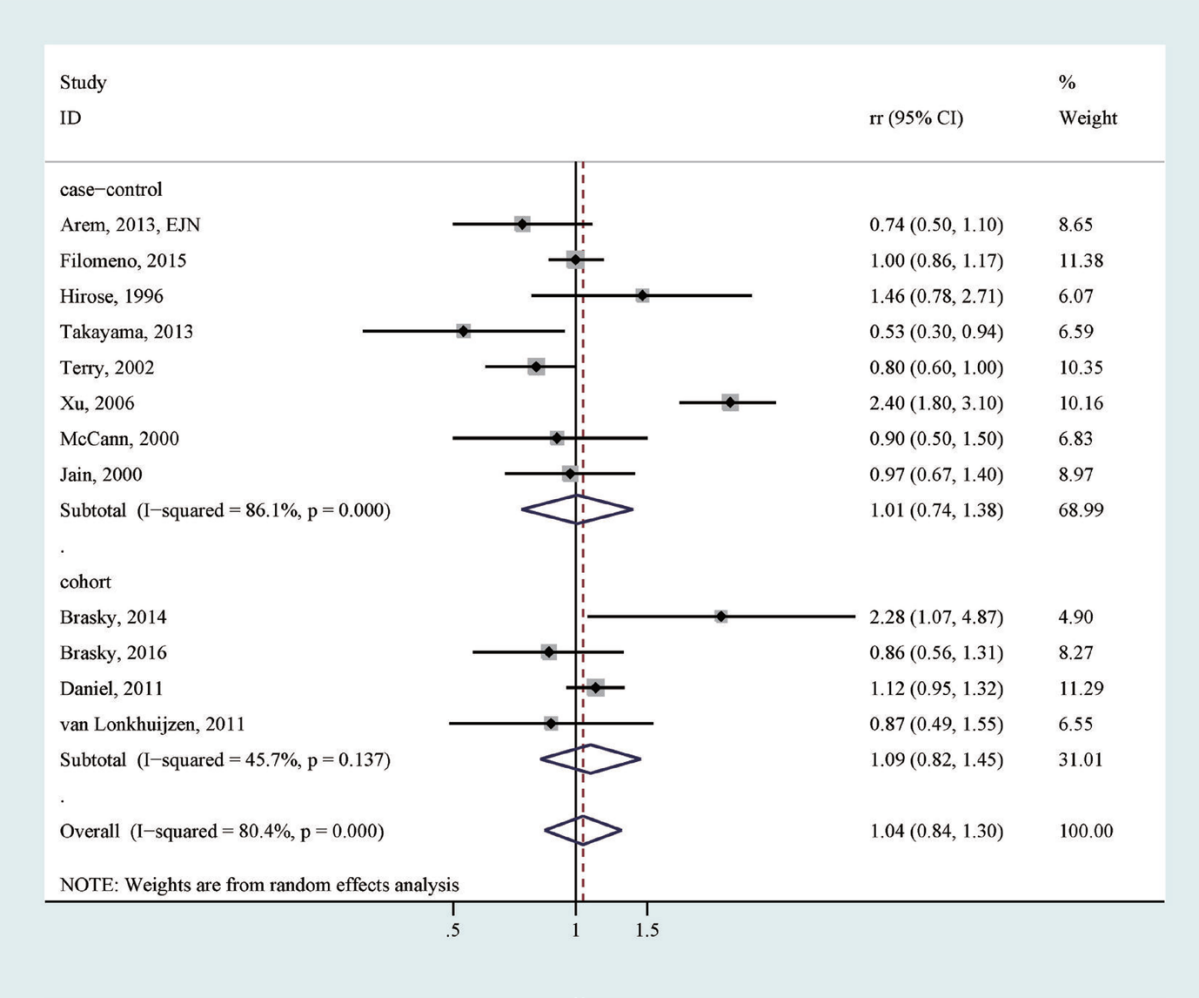
The association between the highest vs. lowest category of fish consumption and endometrial cancer risk

Twelve studies reported the association of fish consumption with endometrial cancer risk comparing the highest category with the lowest category [14–16, 18–22, 24, 25, 28, 31]. After pooling the results of these studies, we did not detect a significant association between the highest *vs*. lowest category of fish consumption and endometrial cancer risk (RR = 1.04, 95% CI = 0.84–1.30), with relatively high heterogeneity (*I*^2^ = 80.4%; Table 3, Figure 2). No apparent publication bias was identified by Egger’s test (*p* for bias: 0.850) or Begg’s test (*p* for bias: 0.631). According to the subgroup analyses (Table 3), the null association persisted in strata according to study design, location, type of controls, number of cases, publication year, and adjustments of energy intake, reproductive factors, and smoking (Table 3).

**Table 3:** Summary risk estimates of the association between fish consumption and endometrial cancer risk (the highest category versus the lowest category)

	No. of reports	RR (95% CI)	*I*^2^	*P* for heterogeneity
**Overall**	12	1.04 (0.84–1.30)	80.4%	< 0.001
**Subgroup analysis**				
**Study design**				
Prospective	4	1.09 (0.82–1.45)	45.7%	0.137
Case-control	8	1.01 (0.74–1.38)	86.1%	< 0.001
**Location**				
North America	7	0.99 (0.82–1.19)	35.3%	0.158
Europe	2	0.92 (0.74–1.13)	53.5%	0.142
Asia	3	1.26 (0.50–3.17)	91.0%	< 0.001
**Type of controls**				
Population-based	6	0.95 (0.60–1.52)	89.7%	< 0.001
Hospital-based	2	1.07 (0.81–1.41)	25.2%	0.248
**Number of cases**				
< 500	6	0.98 (0.69–1.39)	55.8%	0.046
≥ 500	6	1.09 (0.81–1.45)	88.5%	< 0.001
**Study publication time**				
Earlier than 2010	5	1.20 (0.72–1.99)	89.3%	< 0.001
2010–	7	0.95 (0.78–1.15)	57.3%	0.029
**Estimate adjusted for energy intake**				
Yes	8	1.14 (0.88–1.48)	83.6%	< 0.001
No	4	0.84 (0.61–1.17)	47.2%	0.128
**Estimate adjusted for reproductive factors**				
Yes	9	1.14 (0.85–1.54)	81.8%	< 0.001
No	3	0.84 (0.59–1.21)	78.7%	0.009
**Estimate adjusted for smoking**				
Yes	9	0.98 (0.86–1.13)	41.2%	0.092
No	3	1.06 (0.39–2.85)	92.7%	< 0.001
**Estimate adjusted for energy intake, reproductive factors and smoking**				
Yes	5	0.97 (0.78–1.20)	44.0%	0.128
No	7	1.06 (0.74–1.53)	86.8%	< 0.001

Data from eight studies were used to evaluate a non-linear dose-response relationship between fish intake and risk of endometrial cancer risk [13, 15, 16, 18, 22, 25, 28, 31]. Assuming a non-linear relationship, the dose-response analysis suggested a non-significant relationship (*p*: 0.81). The test for nonlinearity suggested that a linear relationship might be more appropriate (*p* for nonlinearity: 0.77). Assuming a linear relationship, we also incorporated two additional studies with available data [29, 30], and detected that the combined RR per one additional serving/week of fish intake was 1.00 (95% CI 0.94–1.07), with considerable heterogeneity (*P* for heterogeneity < 0.001) (Table 4). Subgroup analyses suggested that for every one additional serving/week of fish intake, although the null association existed in the majority of tested strata, a significant inverse association was detected in studies conducted in Europe (RR: 0.90 (0.84–0.97)) (Table 4), and a significant positive association was detected in studies conducted in Asia (RR: 1.15 (1.10–1.21)) (Table 4). Furthermore, studies adjusting for smoking suggested a significant inverse association (RR: 0.95 (0.91–1.00)), while studies without an adjustment of smoking revealed a significant positive association (RR: 1.14 (1.09–1.19)) (Table 4).

**Table 4:** Summary risk estimates of the association between fish consumption and endometrial cancer risk (every one additional serving/week of fish intake)

	No. of reports	RR (95% CI)	*I*^2^	*P* for heterogeneity
**Overall**	10	1.00 (0.94–1.07)	81.7%	< 0.001
**Subgroup analysis**				
**Study design**				
Prospective	2	1.00 (0.97–1.02)	0%	0.778
Case-control	8	1.01 (0.92–1.10)	83.9%	< 0.001
**Location**				
North America	6	1.00 (0.95–1.04)	32.6%	0.191
Europe	2	**0.90 (0.84–0.97)**	0%	0.941
Asia	2	**1.15 (1.10–1.21)**	0%	0.531
**Type of controls**				
Population-based	9	1.02 (0.93–1.12)	83.2%	< 0.001
Hospital-based	1	0.90 (0.80–1.00)	-	-
**Number of cases**				
< 500	5	1.03 (0.97–1.10)	42.5%	0.138
≥ 500	5	0.97 (0.86–1.10)	90.1%	< 0.001
**Study publication time**				
Earlier than 2010	7	1.02 (0.93–1.12)	82.7%	< 0.001
2010–	3	0.98 (0.94–1.03)	19.7%	0.288
**Estimate adjusted for energy intake**				
Yes	5	1.01 (0.93–1.11)	88.2%	< 0.001
No	5	0.99 (0.89–1.10)	72.5%	0.006
**Estimate adjusted for reproductive factors**				
Yes	6	1.01 (0.93–1.09)	85.4%	<0.001
No	4	1.00 (0.89–1.12)	79.1%	0.002
**Estimate adjusted for smoking**				
Yes	6	**0.95 (0.91–1.00)**	41.1%	0.131
No	4	**1.14 (1.09–1.19)**	0%	0.399
**Estimate adjusted for energy intake, reproductive factors and smoking**				
Yes	3	0.98 (0.95–1.02)	19.5%	0.289
No	7	1.02 (0.92–1.12)	82.1%	< 0.001

### Intake of EPA/DHA and endometrial cancer risk

After pooling relevant association estimates from four studies [16–18, 24], a nonsignificant inverse association between the highest category of EPA intake and endometrial cancer risk was detected (RR = 0.79, 95% CI = 0.61–1.04; *I*^*2*^ = 57.7%). Based on dose-response analyses of two studies [17, 24], there was neither a nonlinear relationship (*p*: 0.24), nor a significant linear relationship (*p*: 0.66) of EPA intake.

**Figure 3:**
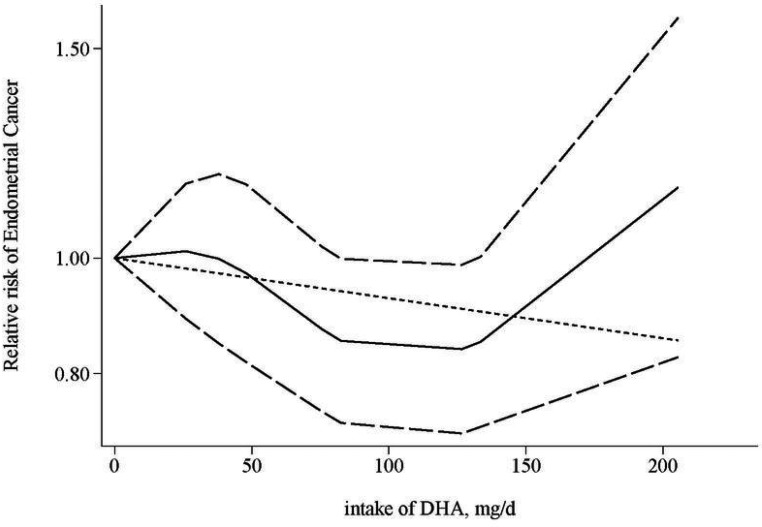
Dose-response relationship for the association between intake of DHA and endometrial cancer risk The solid line represents the estimated relationship. The dashed line represents the 95% confidence interval of the estimated relationship.

Based on four studies [16–18, 24] reporting association of DHA, there was a nonsignificant inverse association between the highest category of DHA intake and endometrial cancer risk (RR = 0.85, 95% CI = 0.64–1.11; *I*^2^ = 59.6%). The dose-response analysis suggested a significant non-linear relationship between DHA intake and endometrial cancer risk (*p*: 0.04; *p* for heterogeneity: 0.39; Figure 3). The test for nonlinearity suggested that such a nonlinear relationship might be more appropriate than a linear relationship (*p* for nonlinearity: 0.04).

## DISCUSSION

We performed a comprehensive systematic review and meta-analysis of epidemiological studies to assess the association between intake of fish/n-3 PUFA and endometrial cancer risk. After summarizing all available evidence, no significant association was detected for intake of overall fish, which is consistent with findings from an earlier meta-analysis [26]. However, a significant inverse association between every one additional serving/week of fish intake and endometrial cancer risk was detected in studies conducted in Europe, and studies adjusted for smoking. With regards to n-3 PUFA, we detected a significant non-linear relationship between DHA intake and risk of endometrial cancer, with a decreased risk being detected for an intake of DHA no more than ∼175 mg/d. A high EPA intake was also suggestively associated with decreased endometrial cancer risk. Further studies would be needed to better characterize the relationship of interest.

The identified inverse association between DHA intake and endometrial cancer risk is plausible from biological perspectives. DHA is associated with reduced inflammation [32–35], which is known to play an important role in endometrial cancer etiology[36–39]. The anti-inflammatory properties of DHA may be due not only to the inhibition of nuclear factor kappa B, but also the downstream modulation of the cyclooxygenase-2 pathway [40]. It has been suggested that the inhibition of the cyclooxygenase-2 blockade may also be associated with reduced estrogen synthesis [41], which is known to be able to drive endometrial proliferation [42, 43]. Furthermore, research suggests that n-3 PUFA may decrease the production of superoxide and free radicals [44], and may influence insulin sensitivity and cell membrane fluidity [45, 46], all of which may protect from carcinogenesis. This is consistent with our finding of an inverse association of high EPA intake, albeit the statistical significance is not reached.

Our finding of a null association of overall fish intake may be explained by several reasons. One possible explanation is that only fatty fish is associated with reduced endometrial cancer risk due to its enrichment of n-3 PUFA, and thus many studies assessing the overall fish intake could not identify the association due to the mixture of fish types evaluated. In a nation-wide case-control study in Sweden, it was indeed that only consumption of fatty fish, but not other types of fish, was significantly associated with reduced endometrial cancer risk. Another reason is that different methods of cooking/preparing fish may induce the heterogeneities across studies. For example, in China, people often tend to cook fish using deep frying methods, which could lead to formation of mutagens and carcinogens, such as heterocyclic amines [16, 29]. Many Chinese population may also consume salted, dried fish which may contains N-nitrosamines. These may explain why studies in Asia (China) detected a positive association between fish intake and endometrial cancer development [16, 25, 29], in contract to studies in other regions such as Europe, which suggested an inverse association. Careful collection and analyses of fish cooking methods, along with fish types may be able to provide a better clue for the heterogeneity of the identified associations in different regions. Furthermore, it is possible that residual confounding may be an issue for some of the included studies. For example, studies adjusting for smoking, a known risk factor for endometrial cancer risk, suggested a significant inverse association for every one additional serving/week of fish intake while those without smoking adjustment suggested the opposite direction.

Our study has several strengths. To our knowledge, this is the most comprehensive meta-analysis evaluating the association between fish consumption and endometrial cancer risk. The synthetization of evidence focusing on n-3 PUFA with endometrial cancer risk, based on our knowledge, is for the first time. In addition to conducting multiple subgroup analyses to further evaluate the association, we performed dose-response analyses to further clarify the relationship. Findings of our analyses suggested a potential beneficial role of DHA/EPA in reducing endometrial cancer development, which warrants further investigation.

Several potential limitations of the present study need to be acknowledged. First, as mentioned above, the fact that we do not have access to the individual level data from the included studies raises a possibility that the association estimates used in our study may not be fully adjusted for. Reproductive factors and smoking are known risk factors for endometrial cancer [43, 47, 48]. However, in some of the studies included in our meta-analyses, not all of these known factors were sufficiently adjusted for [13–15, 19, 29, 30]. Second, differences in the assessment of intake of fish/n-3 PUFA across studies could be an important source of heterogeneity. In seven of the 16 included studies, trained interviewers collected the dietary intake data [13, 20, 22, 25, 29–31], and in the remaining nine studies, a self-administered questionnaire was used to collect dietary information [14–19, 21, 24, 49]. It is known that the collection method of questionnaire may cause inaccuracy and measurement error. Recall bias may also be an issue in the 11 included case-control studies. Additional well-designed studies are warranted to validate our findings. Third, the evidence synthesized from our analyses was from observational studies, which are known to confer biases due to the observational nature. The causal relationship could not be inferred from such studies. Fourth, we noticed considerable heterogeneities across studies in our pooled analyses. We conducted multiple subgroup analyses with the hope of detecting potential factors for such heterogeneities; however, in many strata the heterogeneity remains relatively high. All these limitations need to be considered when interpreting our findings. Fifth, our analyses comparing the highest category versus the lowest category of dietary intake and dose-response analyses did not always suggest the same pattern of findings, especially for analyses of fish intake. For example, the detected significant associations for every one additional serving/week of fish intake in subgroups of studies conducted in Asia or Europe or according to smoking adjustment were not suggested in the analyses comparing the highest versus lowest category of fish consumption. Whether such inconsistences were due to dose-response analysis’s better capturing of exposure pattern or not warrants further exploration.

In conclusion, after summarizing all available evidence from epidemiological studies, intake of fish is significantly associated with reduced endometrial cancer risk in studies adjusted for smoking and those conducted in Europe. DHA tends to be inversely associated with risk of endometrial cancer in a nonlinear relationship. Further well-designed studies are warranted to better characterize the relationship between fish, n-3 PUFA and endometrial cancer development.

## MATERIALS AND METHODS

### Data sources and search strategies

We conducted a literature search of PubMed (MEDLINE) and Embase through March 2017 to identify eligible epidemiological studies. The following search keywords were used: (endometrium OR endometrial) AND (malignancies OR malignancy OR neoplasm OR neoplasms OR cancer OR cancers OR adenoma OR adenomas OR carcinoma OR carcinomas) AND (fatty acid OR docosahexaenoic acid OR eicosapentaenoic acid OR docosapentaenoic acid OR alpha-linolenic acid OR polyunsaturated fatty acid OR omega-3 fatty acid OR n-3 fatty acid OR fish OR fish oil OR seafood). There was no language restriction. We also reviewed the reference lists of the identified articles and related review and meta-analysis articles to identify additional potential studies.

### Study selection

Studies were eligible if they (*i*) were prospective studies or case-control studies, (*ii*) evaluated the association between fish intake or n-3 PUFA intake (EPA or DHA) and endometrial cancer risk, and (*iii*) presented odds ratio (OR), relative risk (RR), or hazard ratio (HR) estimates with 95% confidence intervals (CI) or necessary data for calculation. Studies were included regardless of sample size. If multiple publications regarding the same study were identified, we retained the study with the largest number of cases in our analyses.

### Data extraction and quality assessment

A pair of investigators independently conducted the title/abstract screening, full-text screening, data extraction, and quality assessment. Disagreements were resolved by joint reevaluation, with inputs from other investigators. The data that were extracted from each study included the following: author’s name, publication year, study location, study design, and characteristics of the study sample (sample size, age, categories of fish/n-3 PUFA intake, and association estimates). If there were multiple estimates of the association, we used the estimate with adjustments for the most appropriate covariates.

To assess the quality of included studies, we used the Newcastle-Ottawa Quality Assessment Scale [50]. This scale evaluates various aspects including the population and sampling methods, exposure and outcome collections, and statistical matching/adjustments of the data etc. The quality scores were determined for each study, with a maximum possible score of 9. Studies with scores ≥ 7 were classified as high-quality studies; studies with scores < 7 were classified as low-quality studies.

### Statistical methods

We synthesized association estimates for intake of fish, EPA, and DHA. For each one, pooled estimates comparing the highest category and the lowest category of dietary intake were synthesized. Due to the rarity of endometrial cancer in the general population, ORs and HRs were deemed equivalent to RRs and RRs were used to represent association estimates. The *I*^2^ were used to assess heterogeneity across studies, with a *I*^2^ > 50% suggesting high heterogeneity [51, 52]. The random-effects model was used to pool the log-transformed RR when there was high heterogeneity [53], and the fixed-effects model was used when there was no considerable heterogeneity [54]. We also performed subgroup analyses to examine the robustness of the findings within strata defined by study design, location, control type, number of cases (≥ 500 or < 500), publication year (2010- or before 2010), and adjustments of energy intake, reproductive factors, and smoking. With regard to publication bias, Egger’s test [55] and Begg’s test [56] were performed. A *p* value of less than 0.05 was considered to indicate significant publication bias.

For the dose-response analyses, we explored both non-linear and linear relationships between intake of fish/n-3 PUFA (EPA or DHA) and endometrial cancer risk [57]. The method proposed by Greenland et al. [57] was used to determine study-specific slopes (linear trends) and 95% CIs from the natural logs of the RRs and CIs across categories of intake of fish or n-3 PUFA (EPA or DHA). For this analysis, the number of cases, person-years of non-cases, RRs, and 95% CIs for at least three exposure categories were needed. We assessed the relationship at increments of 1 serving/week for fish consumption and 1 mg/d for n-3 PUFA exposures. When fish intake was reported in unit of g/d, it was converted to serving/week by assuming 1 serving = 100 g. We set the midpoint of each reported category by averaging the lower and upper bounds. In studies where the highest category of intake of fish or n-3 PUFA did not have an upper bound, we assumed a same width of the open ended interval with that of its adjacent interval [58, 59]. We also examined potential nonlinear dose-response relationships using fractional polynomial models with restricted cubic splines and four knots at fixed percentiles (5%, 35%, 65%, and 95%) of the distribution [39]. A likelihood ratio test was performed to determine whether a nonlinear or linear relationship was more appropriate. All statistical analyses were performed using Stata 12.1 software (StataCorp, College Station, TX, USA).

## SUPPLEMENTARY MATERIALS TABLE




