# Supplementary material for: Dietary n-3 polyunsaturated fatty acids, fish consumption, and endometrial cancer risk: a meta-analysis of epidemiological studies
Source: Oncotarget. 2017 May 30;8(53):91684–93. doi: 10.18632/oncotarget.18295 (PMC5710957; doi:10.18632/oncotarget.18295)
Supplement: Supplementary file 1 [file oncotarget-08-91684-s001.pdf]

Dietary n-3 polyunsaturated fatty acids, fish consumption, and endometrial cancer risk: a meta-analysis of epidemiological studies

Supplementary Materials

Supplementary Table 1: Characteristics of studies assessing associations of fish/n-3 fatty acids consumption and endometrial cancer risk. See Supplementary_Table_1.
