# Supplementary material for: Dietary n-3 polyunsaturated fatty acids, fish consumption, and endometrial cancer risk: a meta-analysis of epidemiological studies
Source: Oncotarget. 2017 May 30;8(53):91684–93. doi: 10.18632/oncotarget.18295 (PMC5710957; doi:10.18632/oncotarget.18295)
Supplement: Supplementary file 2 [file oncotarget-08-91684-s002.docx]

**Supplementary Table 1: Characteristics of studies assessing associations of fish/n-3 fatty acids consumption and endometrial cancer risk**

| **Author, publication year, location** | **Study type** | **Cases/subject or control (age), duration of follow up** | **Consumption categories (exposure/case assessment)** | **RR (95%CI)** | **Matched/Adjusted factors** |
| --- | --- | --- | --- | --- | --- |
| Arem, 2013, Connecticut, US | PC-CS | 556/533 (mean ~61) | Total fish:  0-0.46 serving/week  0.46-1.04 servings/week  1.04-2 servings/week  ≥2 servings/week  Every increase of 1 serving/week  EPA:  quartile 1  quartile 2  quartile 3  quartile 4  DHA:  quartile 1  quartile 2  quartile 3  quartile 4  (self-administrated FFQ/hospital visit to obtain case eligibility and identifying information) | 1.0 (ref)  1.05 (0.73–1.52)  1.00 (0.69–1.45)  0.74 (0.50–1.10)  0.93 (0.86–1.02)  1.0 (ref)  0.81 (0.57–1.16)  0.98 (0.69–1.40)  0.57 (0.39–0.84)  1.0 (ref)  0.80 (0.56–1.15)  0.83 (0.58–1.19)  0.64 (0.44–0.94) | energy consumption, age, BMI, number of live  births, menopausal status, oral contraceptive use, hypertension, smoking status, and race/ethnicity |
| Brasky, 2014, western Washington State, US | CS | 263/22,494 (50-76), median 9 years | Total fish:  0 serving/week  0.01-1 serving/week  1.01-2.1 servings/week  2.11-3.08 servings/week  ≥3.09 servings/week  EPA:  quintile 1 (≤20 mg/d)  quintile 2 (21-38 mg/d)  quintile 3 (39–60 mg/d)  quintile 4 (61–105 mg/d)  quintile 5 (>105 mg/d)  DHA:  quintile 1 (≤43 mg/d)  quintile 2 (44-75 mg/d)  quintile 3 (76–118 mg/d)  quintile 4 (119–191 mg/d)  quintile 5 (>191 mg/d)  (FFQ/link with cancer registry, ascentain through hospitals and state death certificates) | 1.0 (ref)  1.46 (0.70, 3.06)  1.36 (0.65, 2.83)  1.54 (0.70, 3.37)  2.28 (1.07, 4.87)  1.0 (ref)  1.19 (0.78, 1.81)  1.05 (0.67, 1.64)  1.16 (0.75, 1.80)  1.23 (0.80, 1.89)  1.0 (ref)  0.98 (0.63, 1.52)  1.21 (0.79, 1.84)  0.93 (0.59, 1.46)  1.35 (0.89, 2.06) | age, race, education, BMI, pack-years of smoking, physical activity, alcohol consumption, age at menarche, age at first birth, age at menopause, parity, years of combined hormone therapy, years of estrogen-only therapy, years of oral contraceptive use, oophoerectomy, family history of uterine cancer, family history of ovarian cancer, history of diabetes, and total energy |
| Brasky, 2016, US | CS | 282/47,602 (21-69), median 18 years | Total fish:  quartile 1  quartile 2  quartile 3  quartile 4  EPA:  quintile 1  quintile 2  quintile 3  quintile 4  quintile 5  DHA:  quintile 1  quintile 2  quintile 3  quintile 4  quintile 5  (FFQ/questionnaire and cancer registry, ascentain through medical records and death certificates) | 1.0 (ref)  0.83 (0.54, 1.26)  1.19 (0.80, 1.76)  0.86 (0.56, 1.31)  1.0 (ref)  0.66 (0.42, 1.05)  0.77 (0.50, 1.20)  0.84 (0.55, 1.29)  0.72 (0.47, 1.10)  1.0 (ref)  1.01 (0.64, 1.60)  0.69 (0.42, 1.13)  1.17 (0.76, 1.78)  0.84 (0.54, 1.30) | age, time period, total energy intake, US region, education, BMI, physical activity, alcohol consumption, smoking, fruit consumption, vegetable consumption, age at menarche, age at menopause, parity, age at first birth, duration of combined hormone therapy, duration of estrogen-alone hormone therapy, duration of oral contraceptive use, and diabetes |
| Daniel, 2011, US | CS | 1,593/492,186 (50-71), mean 9.1 years | Fish:  quintile 1  quintile 2  quintile 3  quintile 4  quintile 5  (FFQ/link to cancer registry, ascentain through SSA Death Master File and National Death Index Plus) | 1.0 (ref)  1.04 (0.88-1.22)  0.97 (0.83-1.15)  1.15 (0.98-1.35)  1.12 (0.95-1.32) | red meat intake, age, sex, education, marital status, family history of cancer, race, BMI, smoking status, frequency of vigorous physical activity, MHT in women, and intake of alcohol, fruit, vegetables, total energy, intake of poultry |
| Filomeno, 2015, Italy and Swiss Canton of Vaud | HC-CS | 1,411/3,668 (median 57) | Fish:  Low  High  (Trained interviewer/histology confirmation) | 1.0 (ref)  1.00 (0.86-1.17) | age, study centre, year of interview, education, tobacco smoking, BMI, age at menopause, age at menarche, parity, oral contraceptive use, hormone-replacement therapy use, history of hypertension, diabetes  and total energy intake |
| Hirose, 1996, Nagoya, Japan | HC-CS | 145/26,751 (20+) | Fish:  ≤3/month  >1-2/week  (self-administrated questionnaire/link to hospital cancer registry, histological diagnosis) | 1.0 (ref)  1.46 (0.78-2.71) | age, first visit year, age at first full-term pregnancy, no. of births, BMI, smoking, physical activity, type of breakfast, milk, raw vegetables, fruit, dietary control, bean curd, green vegetables, carrot, pumpkin, egg |
| Takayama, 2013, Miyagi Prefecture, Japan | PC-CS | 161/380 (mean 54) | Fish:  quartile 1  quartile 2  quartile 3  quartile 4  (FFQ/histology confirmation) | 1.0 (ref)  0.56 (0.32–0.98)  0.58 (0.33–1.02)  0.53(0.30–0.94) | BMI, diabetes history and hypertension history |
| Terry, 2002, Sweden | PC-CS | 709/2,888 (50-74) | Fish:  quartile 1  quartile 2  quartile 3  quartile 4  (questionnaire/link to cancer registry and review of histological specimens) | 1.0 (ref)  1.0 (0.8–1.2)  0.8 (0.6–1.0)  0.8 (0.6–1.0) | age, BMI, smoking, leisure time physical activity, consumption of alcohol, multivitamin use, and prevalence of diabetes |
| van Lonkhuijzen, 2011, Alberta, Toronto, and Western Ontario, Canada | Case cohort | 107/1,830 (mean ~59), average 6.5 years for cases and 11.7 years for subcohort | Fish:  <13.1 g/day  13.1-25.79 g/day  25.79-36.03 g/day  ≥36.03 g/day  (FFQ/link to cancer registry) | 1.0 (ref)  0.66 (0.36-1.20)  0.89 (0.51-1.54)  0.87 (0.49-1.55) | province of recruitment, age, BMI, age at menarche, number of live births, breastfeeding, number of years of oral contraceptive use, exercise, Kcal intake, consumption of cruciferous vegetables, postmenopausal status at baseline, and hormone replacement therapy |
| Xu, 2006, Shanghai, China | PC-CS | 1,204/1,212 (30-69) | Fish:  quartile 1  quartile 2  quartile 3  quartile 4  (trained interviewer/link to cancer registry, confirmed by histopathology or by medical history review) | 1.0 (ref)  1.7 (1.3 – 2.2)  1.9 (1.4 – 2.4)  2.4 (1.8 – 3.1) | age, menopausal status, diagnosis of diabetes, alcohol consumption, BMI, physical activity, and total energy intake |
| McCann, 2000, Western Ney York, US | PC-CS | 232/639 (40-85) | Fish/seafood (monthly frequency):  quartile 1 (<4.5)  quartile 2 (4.6-6.5)  quartile 3 (6.6-9.5)  quartile 4 (>9.5)  (trained interviewer/histology confirmed) | 1.0 (ref)  1.0 (0.6-1.5)  0.9 (0.6-1.5)  0.9 (0.5-1.5) | county of residence, age, education, BMI, diabetes, hypertension, pack-years cigarette smoking, age at menarche, parity, oral contraceptive use, menopause status, postmenopausal estrogen use, intake of fruit, vegetables, red meat, poultry, processed meats, total meat, snacks and sweets, Breads, cereals, rice and pasta, dairy, fats |
| Jain, 2000, Toronto, Halton, Peel, York, Canada | PC-CS | 552/562 (30-79) | Fish (g/d):  quartile 1 (<7.6)  quartile 2 (7.6-18.3)  quartile 3 (18.3-35.6)  quartile 4 (>35.6)  (trained interviewer /link to cancer registry & pathology reports, histology confirmation) | 1.0 (ref)  1.09 (0.77-1.55)  1.02 (0.71-1.47)  0.97 (0.67-1.40) | location, total energy, age, body weight, ever smoked, history of diabetes, used oral contraceptives, used hormone replacement therapy, university education, live births, age at menarche |
| Goodman, 1997, Hawaii, US | PC-CS | 322/511 (18-84) | Fish:  For every one more serving/week  (trained interviewer/link to tumor registry, histologically confirmed) | 1.11 (1.00-1.22) | age, ethnicity |
| Shu, 1993, Shanghai, China | PC-CS | 268/268 (18-74) | Fish:  For every one more serving/week  (trained interviewer/link to cancer registry, histopathologically confirmed) | 1.11 (0.98-1.26) | age |
| Fernandez, 1999, northern Italy | HC-CS | 750/7990 (<75) | Fish:  <1 serving/week  1 serving/week  ≥2 servings/week  Increment 1 serving/week  (trained interviewer/ histologically confirmed) | 1.0 (ref)  0.6 (0.5-0.8)  0.8 (0.6-0.9)  0.9 (0.8-1.0) | age, sex, area of residence, education, smoking, alcohol consumption, BMI |
| Brasky, 2015, US | CS | 1,253/87,360 (50-79), median 13 years | EPA:  quintile 1 (≤15.6 mg/d)  quintile 2 (15.7-27.4 mg/d)  quintile 3 (27.5–41.3 mg/d)  quintile 4 (41.4–63.6 mg/d)  quintile 5 (>63.6 mg/d)  DHA:  quintile 1 (≤32.8 mg/d)  quintile 2 (32.9-51.9 mg/d)  quintile 3 (52.0–76.8 mg/d)  quintile 4 (76.9–121.0 mg/d)  quintile 5 (>121.0 mg/d)  (FFQ/medical record reviewed) | 1.0 (ref)  1.09 (0.89, 1.33)  0.96 (0.78, 1.18)  1.00 (0.81, 1.22)  0.81 (0.65, 1.01)  1.0 (ref)  0.91 (0.74, 1.11)  0.97 (0.79, 1.18)  0.81 (0.66, 1.00)  0.77 (0.63, 0.95) | age, total energy, clinical trial/observational study intervention assignment, US region, race, education, BMI, smoking, alcohol, physical activity, age at menarche, age at first birth, age at menopause, parity, duration of combined menopausal hormone therapy, duration of estrogen-alone hormone therapy,  duration of oral contraceptive use, oophoerectomy status, family history of endometrial cancer, and history of diabetes |

CS: cohort study; PC-CS: population-based case-control study; HC-CS: hospital-based case-control study; FFQ: food frequency questionnaire; RR: relative risk; CI: confidence interval; Ref: reference; N/A: not available; BMI: body mass index.
